# Sex‐ and tissue‐specific changes in mTOR signaling with age in C57BL/6J mice

**DOI:** 10.1111/acel.12425

**Published:** 2015-11-24

**Authors:** Emma L. Baar, Kathryn A. Carbajal, Irene M. Ong, Dudley W. Lamming

**Affiliations:** ^1^ Department of MedicineUniversity of Wisconsin‐MadisonMadisonWIUSA; ^2^ William S. Middleton Memorial Veterans HospitalMadisonWIUSA; ^3^ Department of Biostatistics and Medical InformaticsUniversity of Wisconsin‐MadisonMadisonWIUSA; ^4^ University of Wisconsin Carbone Comprehensive Cancer CenterUniversity of WisconsinMadisonWIUSA

**Keywords:** aging, mice, mTOR, mTORC1, mTORC2, rapamycin, sexual dimorphism

## Abstract

Inhibition of the mTOR (mechanistic Target Of Rapamycin) signaling pathway robustly extends the lifespan of model organisms including mice. The precise molecular mechanisms and physiological effects that underlie the beneficial effects of rapamycin are an exciting area of research. Surprisingly, while some data suggest that mTOR signaling normally increases with age in mice, the effect of age on mTOR signaling has never been comprehensively assessed. Here, we determine the age‐associated changes in mTORC1 (mTOR complex 1) and mTORC2 (mTOR complex 2) signaling in the liver, muscle, adipose, and heart of C57BL/6J.Nia mice, the lifespan of which can be extended by rapamycin treatment. We find that the effect of age on several different readouts of mTORC1 and mTORC2 activity varies by tissue and sex in C57BL/6J.Nia mice. Intriguingly, we observed increased mTORC1 activity in the liver and heart tissue of young female mice compared to male mice of the same age. Tissue and substrate‐specific results were observed in the livers of HET3 and DBA/2 mouse strains, and in liver, muscle and adipose tissue of F344 rats. Our results demonstrate that aging does not result in increased mTOR signaling in most tissues and suggest that rapamycin does not promote lifespan by reversing or blunting such an effect.

## Introduction

Rapamycin is an FDA‐approved compound that robustly extends the lifespan of yeast, worms, flies, and mice (Powers *et al*., 2006; Bjedov *et al*., 2010; Miller *et al*., 2011; Robida‐Stubbs *et al*., 2012). Rapamycin is an acute inhibitor of mTORC1 (mechanistic Target Of Rapamycin Complex 1), which regulates numerous cellular processes including ribosomal biogenesis, protein translation, and autophagy through the phosphorylation of substrates that include S6K1, 4E‐BP1, and Ulk1 (Laplante & Sabatini, 2012). Mice lacking *S6K1* or with decreased mTORC1 activity have extended lifespan, demonstrating that decreased mTORC1 signaling is sufficient to promote longevity, at least in females (Selman *et al*., 2009; Lamming *et al*., 2012). A similar inverse correlation between hepatic mTORC1 signaling and lifespan is also observed in wild‐type mice (Solon‐Biet *et al*., 2014).

One conceptual model to explain the beneficial effect of rapamycin on lifespan is that rapamycin may act by counteracting an age‐associated increase in mTORC1 signaling. Alternatively, mTORC1 signaling may be optimal during development, but inappropriately high for the maintenance of health later in life, and rapamycin acts to repress this overactive mTORC1 signaling (Blagosklonny, 2009). In both of these conceptual models, mTORC1 signaling in the aged may drive much of the pathophysiology of aging. While it is not clear that increased mTORC1 signaling inevitably accelerates aging, increased mTORC1 signaling is associated with numerous age‐related diseases and pathologies, including Alzheimer's disease (An *et al*., 2003), diabetes (Inoki *et al*., 2011; Volkers *et al*., 2014), and cancer (Bar‐Peled *et al*., 2013; Grabiner *et al*., 2014). Increased mTORC1 signaling is found in human cells and certain animal models of Hutchinson–Gilford progeria syndrome, a rare, fatal genetic disorder characterized by the early onset of conditions associated with old age (Cao *et al*., 2011; Ramos *et al*., 2012). Conversely, dietary regimens that extend lifespan, such as calorie restriction and protein restriction, are associated with reduced mTORC1 signaling (Lamming & Anderson, 2014; Solon‐Biet *et al*., 2014; Lamming *et al*., 2015).

Collectively, these studies demonstrate that mTORC1 signaling is inversely correlated with mouse lifespan and that genetic mutations which increase mTORC1 signaling can result in the development of age‐associated pathologies. The question of what happens to mTORC1 signaling during normal aging has therefore received significant attention. As reported in Table 1, numerous studies have reported increased mTORC1 signaling with age in HSCs (hematopoietic stem cells), hypothalamic POMC neurons, and in the liver and lung of mice (Chen *et al*., 2009; Sengupta *et al*., 2010; Yang *et al*., 2012; Leontieva *et al*., 2014; Calhoun *et al*., 2015).

**Table 1:** Reported changes in mTORC1 signaling with age. The direction of mTORC1 signaling, with the tissues, ages, substrates, species, sex and strain (where available) are specified

mTORC1 signaling	Tissue(s)	Ages	Substrate(s)	Species/strain	Sex and feeding status	Reference
Up	Aorta	6, 30 and 36 months	S6K1	Rat; F344/BNF1	Male; *ad libitum*	Rice *et al*. (2005)
Down	Adipose; Heart	4 vs. 28 months	Transcriptional profiling	Rat; F344	Male; NS	Linford *et al*. (2007)
Up	HSCs	2 vs. 26 months	mTOR; S6K1; S6	Mouse; C57BL/6J.Nia	NS; NS	Chen *et al*. (2009)
Up	Liver	2–8 vs. 20–24 months	S6	Mouse; C57BL/6	NS; Fasted	Sengupta *et al*. (2010)
Down	Liver; Muscle	6 vs. 24 months	S6K1	Mouse; C57BL/6JRj	NS; Fasted	Houtkooper *et al*. (2011)
Down	Blood	20–102 years	Transcriptional profiling	Human	Male and female; Fasted	Harries *et al*. (2012)
Up	POMC neurons	1 vs. 12 months	S6	Mouse; C57BL/6	Male; NS	Yang *et al*. (2012)
Up	Liver	10 vs. 28 months	S6	Mouse; C57BL/6NCr	Female; Fasted	Leontieva *et al*. (2014)
Up	Lung	8, 24, and 32 months	mTOR; S6K1	Mouse; C57BL/6J.Nia	Male; NS	Calhoun *et al*. (2015)

HSCs, hematopoietic stem cells; NS, not stated in the reference.

However, the literature is not unanimous in this conclusion (Table 1). Surprisingly, Houtkooper *et al*. (2011) determined that mTORC1 signaling in liver and skeletal muscle decreases during aging. While this study stands alone in reporting a decrease in mTORC1 substrate phosphorylation during aging, it is supported by microarray data from rats which identified a transcriptional downregulation of mTOR signaling with aging in the heart and adipose tissue of F344 (Fischer 344) rats (Linford *et al*., 2007). Transcriptional profiling of human blood likewise identified an age‐associated decrease in mTOR signaling (Harries *et al*., 2012).

Our reading of the literature suggested that several of the studies in which mTOR signaling was reported to increase with age utilized very young mice or utilized a broad range of ages. Many studies also suffered from a lack of subjects and were only able to examine the phosphorylation status of a single mTORC1 substrate in a single tissue. Sex and genetic backgrounds were also not clearly specified in all studies.

To better understand how mTOR signaling changes during aging in wild‐type animals, we examined the liver, skeletal muscle, heart, and adipose tissue of male and female C57BL/6J mice from the NIA (National Institute on Aging) Aged Rodent Colony. Young (6 month), Middle‐aged (22‐month female/24‐month male), and Old (26‐month female/30‐month male) mice were used. We examined signaling downstream of mTORC1 as well as mTORC2. Finally, we also examined mTOR signaling in the livers of HET3 mice (Lamming *et al*., 2013) as well as available tissues from DBA/2 inbred mice and F344 rats obtained from the NIA Aged Rodent Tissue Bank. We find that the trajectory of mTOR signaling with age varies by tissue, gender, and feeding status, as well as the precise substrate examined. Strikingly, while mTORC1 signaling does increase with age in some contexts, we do not observe a generalized increase in mTORC1 or mTORC2 signaling with age.

## Results

### mTORC1 signaling decreases with age in liver, but increases with age in adipose tissue

We conducted our study of mTOR signaling in mouse aging in C57BL/6J.Nia mice from the NIA Aged Rodent Colony, a widely used mouse resource. Importantly, the lifespan of these mice can be extended by rapamycin treatment (Fok *et al*., 2014), and aging has been reported to elevate mTOR signaling in HSCs (Chen *et al*., 2009) and the lungs (Calhoun *et al*., 2015) of these mice (Table 1). Lifespan curves of mice in the NIA Aged Rodent Colony have been published (Turturro *et al*., 1999), and we selected the following ages to analyze: a ‘Young’ group of adult male and female mice at 6 months of age (near 100% survival for both males and females); a ‘Middle’‐aged group of 22‐month‐old females and 24‐month‐old males (approximately 70% survival), and an ‘Old’ group of 26‐month‐old females and 30‐month‐old males (approximately 35% survival).

**Figure 1:**
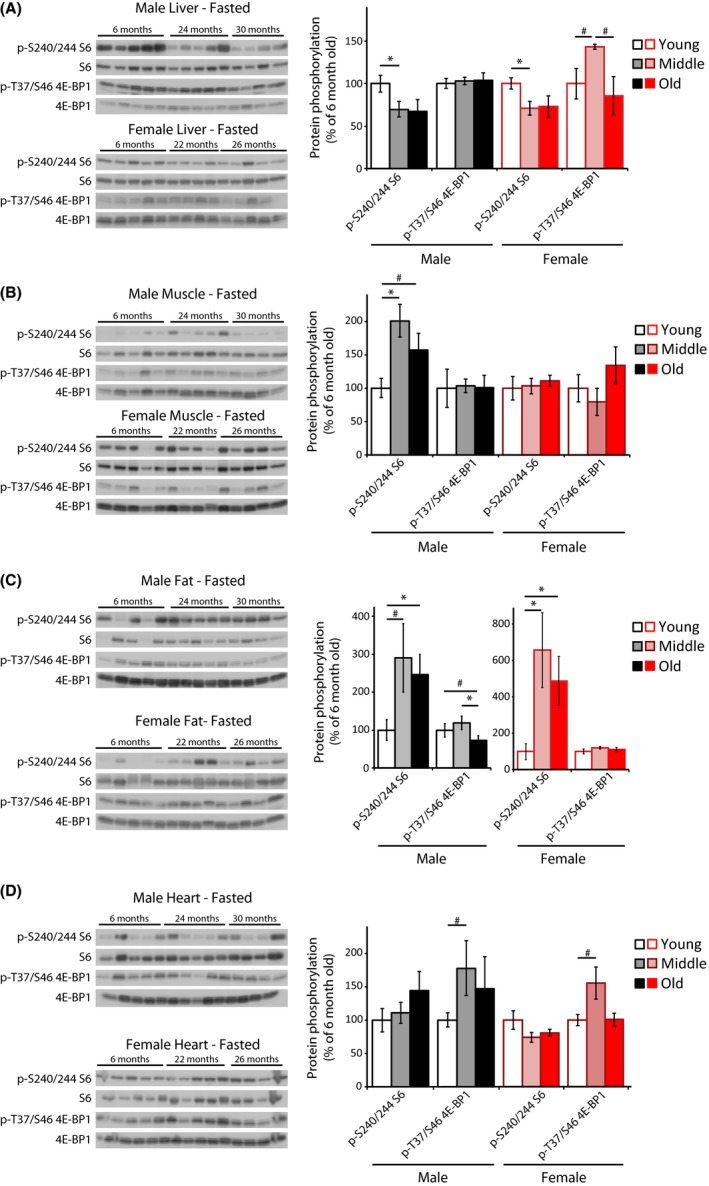
mTORC1 activity in fasted male and female C57BL/6J.Nia mice. (A–D) The phosphorylation of S6 and 4E‐BP1 was assessed by Western blotting of (A) liver, (B) muscle, (C) adipose, and (D) heart tissue lysates. Additional western blots included in the quantification are shown in Fig. S1A. Young refers to 6‐month‐old males and females (10 males, 5 females), Middle refers to 24‐month‐old males and 22‐month‐old females (10 males, 5 females), and Old refers to 30‐month‐old males and 26‐month‐old females (8 males, 4 females). Quantification of S6 S240/S244 is relative to S6, and 4E‐BP1 T37/S46 is relative to 4E‐BP1 (*P < 0.05, #*P *< 0.09, two‐tailed *t*‐test, error bars indicate standard error).

As mTOR is activated by nutrients and growth factor signaling, we decided to initially analyze mice after an overnight fast in order to assess basal levels of mTOR signaling. We first analyzed mTORC1 signaling in liver (Fig. 1A) and skeletal muscle (Fig. 1B), due to the conflicting previous reports (Sengupta *et al*., 2010; Houtkooper *et al*., 2011; Leontieva *et al*., 2014). In agreement with the results seen by Houtkooper *et al*., we observed a 30% decrease in S6 S240/S244 phosphorylation in the livers of Middle‐aged males and females compared to Young (Fig. 1A), with a comparable trend in ‘Old’ mice. However, we observed a doubling of S6 phosphorylation in the skeletal muscle of Middle‐aged and Old males (Fig. 1B). Interestingly, this result was limited to males, and no change was observed in females.

We proceeded to also analyze mTORC1 signaling in white adipose tissue and heart, tissues in which mTOR signaling has been predicted to decrease with age based on transcriptional profiling (Linford *et al*., 2007). Surprisingly, we observed a massive increase in S6 phosphorylation in adipose tissue, with phosphorylation of S6 increasing three‐fold in Middle‐aged and Old males and approximately six‐fold in Middle‐aged and Old females (Fig. 1C). We observed no effect of age on S6 phosphorylation in heart tissue (Fig. 1D).

In all four of these tissues, we also analyzed phosphorylation of 4E‐BP1 T37/S46, a direct mTORC1 substrate. We observed only minor effects of aging on this substrate, although there was a trend (*P *< 0.09) toward increased 4E‐BP1 T37/S46 phosphorylation in Middle‐aged (but not Old) female liver and heart (Fig. 1A,D), and a trend (*P *< 0.08) toward a 25% decrease in 4E‐BP1 T37/S46 phosphorylation in Old male adipose tissue (Fig. 1C).

### Increased mTORC2 signaling in fasted male mice correlates with increased fasting insulin

One of the primary regulators of mTORC1 activity is Akt, which activates mTORC1 activity through the inhibitory phosphorylation of TSC2 and PRAS40 (Laplante & Sabatini, 2012). Genetic studies in worms, flies, and mice consistently find that decreased signaling through the PI3K/Akt/mTORC1 signaling axis promotes longevity (Lamming, 2014), and calorie restriction, an intervention that promotes longevity in numerous species, decreases PI3K/Akt signaling in humans (Mercken *et al*., 2013).

The mTOR protein kinase is found in two complexes, mTORC1, discussed above, and mTORC2, which regulates a diverse set of substrates including specific residues of Akt (Sarbassov *et al*., 2006; Lamming, 2014). We and others have found that chronic treatment with rapamycin inhibits not only mTORC1, but also inhibits mTORC2 in numerous tissues, including liver, muscle, adipose tissue, and heart (Lamming *et al*., 2012; Schreiber *et al*., 2015). The role of mTORC2 in aging is not yet clear, as mice with decreased expression of *Rictor*, an essential component of mTORC2, have a male‐specific decrease in lifespan, while mice heterozygous for *Akt1* have increased lifespan (Nojima *et al*., 2013; Lamming *et al*., 2014b).

**Figure 2:**
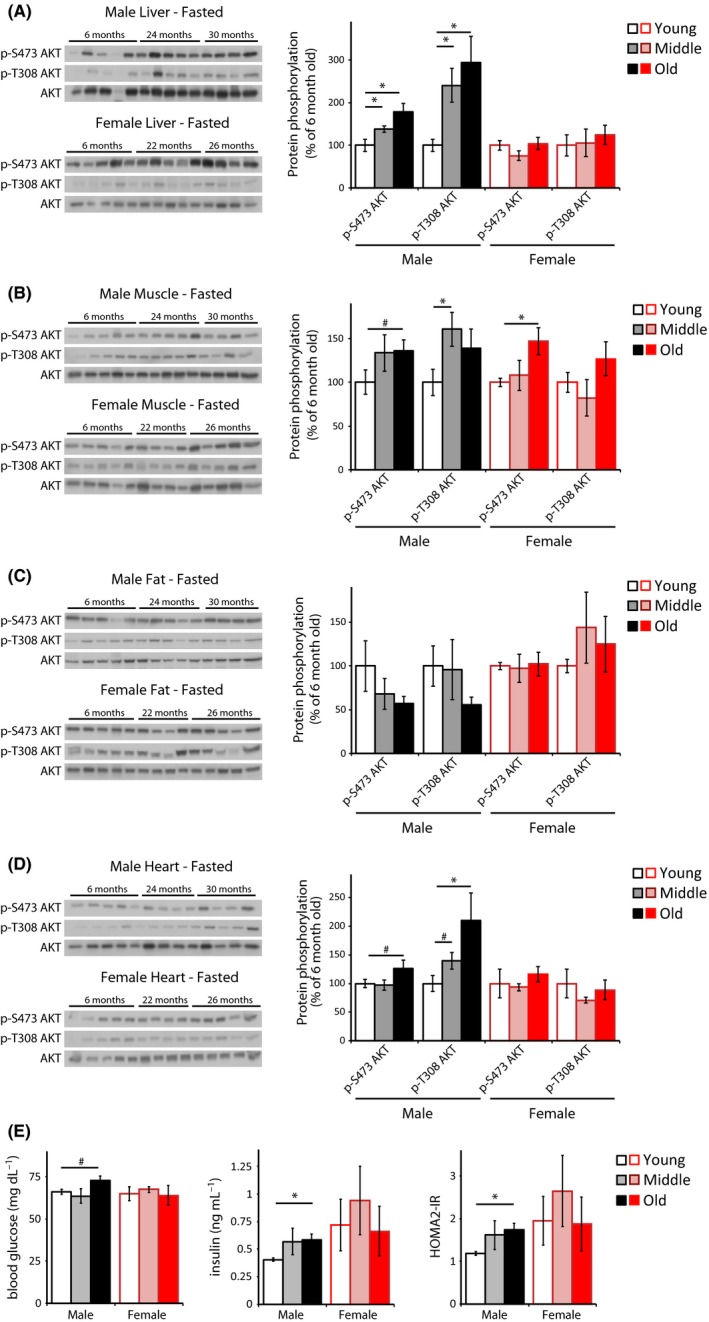
mTORC2 activity in fasted male and female C57BL/6J.Nia mice. (A–D) The phosphorylation of AKT was assessed by Western blotting of (A) liver, (B) muscle, (C) adipose, and (D) heart tissue lysates. Additional western blots included in the quantification are shown in Fig. S1B. Young refers to 6‐month‐old males and females (10 males, 5 females), Middle refers to 24‐month‐old males and 22‐month‐old females (10 males, 5 females), and Old refers to 30‐month‐old males and 26‐month‐old females (8 males, 4 females). Quantification of AKT T308 and S473 are relative to AKT (*P < 0.05, #*P *< 0.09, two‐tailed *t*‐test, error bars indicate standard error). (E) Fasting blood glucose and insulin levels were assessed in Young, Middle and Old male and female mice, and HOMA2‐IR was calculated (males: *n *= 5 Young, 5 Middle, 5 Old; females: *n *= 7 Young, 7 Middle, 6 Old; *P = < 0.05, #*P *= 0.054, two‐tailed *t*‐test).

We analyzed the age‐associated phosphorylation of two specific residues on AKT, S473 (a direct mTORC2 substrate) and T308 (a PDK1 substrate) in liver, muscle, adipose tissue, and heart (Fig. 2). We observed an age‐associated increase in the phosphorylation of both AKT residues in the liver, muscle, and heart of fasted male mice (Fig. 2A,B,D) but not in adipose (Fig. 2C). With the exception of increased AKT S473 phosphorylation in the muscle of Old female mice (Fig. 2B), we observed no change in the phosphorylation of these residues with age in female mice.

To determine the contribution of physiological insulin signaling to these effects on AKT phosphorylation, we analyzed fasting glucose and insulin levels in both male and female mice (Fig. 2E). We observed an age‐associated increase in fasting insulin levels in males, as well as fasting hyperglycemia in Old male mice, but no change in fasting glucose or insulin levels with age in females (Fig. 2E). Thus, the age‐associated increase in the phosphorylation of AKT T308 and S473 in male liver, skeletal muscle, and heart is largely consistent with increased levels of insulin, but does not explain the increased AKT S473 phosphorylation observed in aged female muscle (Fig. 2B). Analysis of other hormonal effectors of PI3K signaling, such as IGF‐1 and leptin, could also impact AKT phosphorylation and would be interesting to pursue in a future study.

### Age‐associated changes in mTOR signaling vary following refeeding

As mTOR signaling is nutrient sensitive, it can be observed not only in the basal fasted state, but also after nutrient stimulation. We have previously utilized a paradigm in which mice are fasted overnight and then refed for 45 min prior to sacrifice and tissue harvest to reveal differences in mTORC1 and mTORC2 signaling (Lamming *et al*., 2012, 2014a).

**Figure 3:**
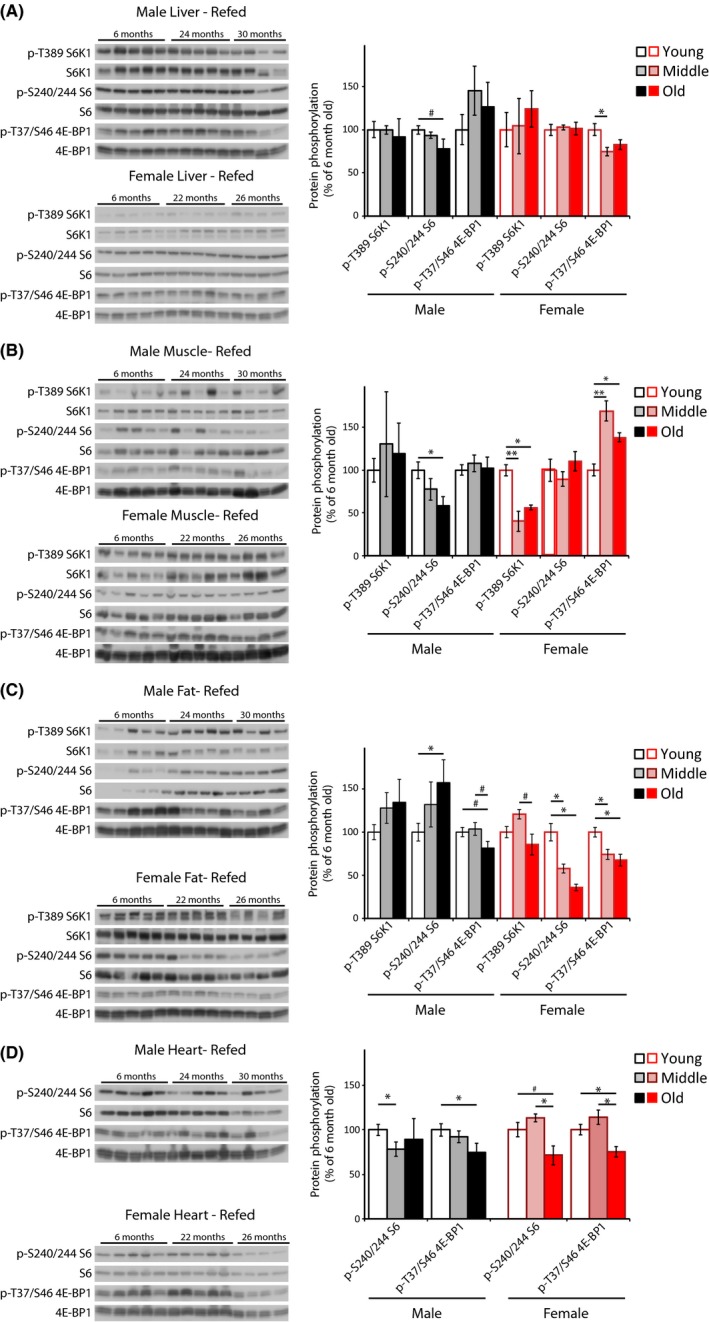
mTORC1 activity in fed male and female C57BL/6J.Nia mice. (A–D) The phosphorylation of S6K1, S6, and 4E‐BP1 was assessed by Western blotting of (A) liver, (B) muscle, (C) adipose, and (D) heart tissue lysates from mice that were fasted overnight and then refed for 45 min. Additional western blots included in the quantification are shown in Fig. S2A. Young refers to 6‐month‐old males and females (10 males, 5 females), Middle refers to 24‐month‐old males and 22‐month‐old females (10 males, 5 females), and Old refers to 30‐month‐old males and 26‐month‐old females (8 males, 4 females). Quantification of S6K1 T389 is relative to S6K1, S6 S240/S244 is relative to S6, and 4E‐BP1 T37/S46 is relative to 4E‐BP1 (**P < 0.01, *P < 0.05, #*P *< 0.09, two‐tailed *t*‐test, error bars indicate standard error).

As shown in Fig. 3, minor decreases in S6 and 4E‐BP1 phosphorylation in liver are observed in refed aged males and females, respectively (Fig. 3A). In contrast to the fasted state, S6 phosphorylation is decreased in refed aged male muscle, and decreased S6K1 T389 phosphorylation is observed in refed aged female liver (Fig. 3B). While S6 phosphorylation was massively increased with age in fasted females, after refeeding, aged females display decreased S6 phosphorylation (Fig. 3C). We also observed a decrease in S6 phosphorylation in both refed aged male and female heart (Fig. 3D).

In contrast to the fasted state, sharp differences in 4E‐BP1 phosphorylation emerge with aging in the refed state. Decreased 4E‐BP1 T37/S46 phosphorylation with age is observed in female liver, fat, and heart tissue, as well as in male fat and heart tissue. Only in skeletal muscle does female 4E‐BP1 T37/S46 phosphorylation increase with age (Fig. 3B). These results, as for S6 phosphorylation, are largely consistent with decreased mTORC1 signaling in aged mice following refeeding.

**Figure 4:**
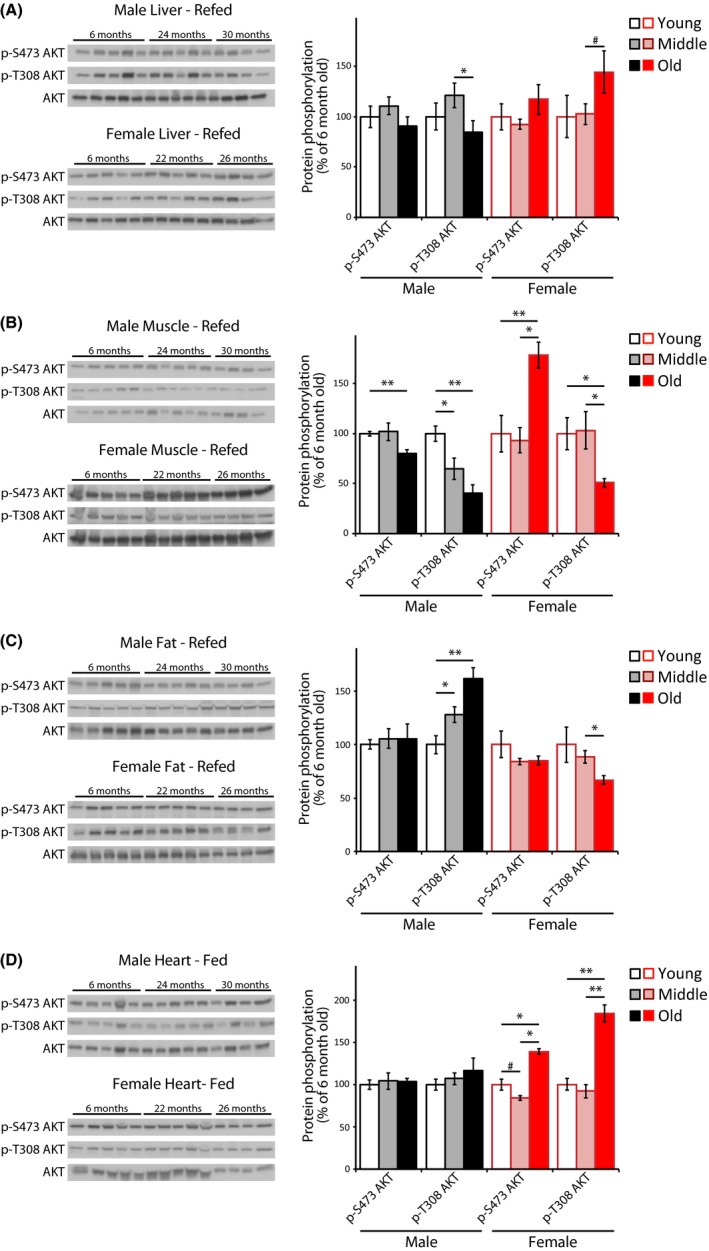
mTORC2 activity in refed male and female C57BL/6J.Nia mice. (A–D) The phosphorylation of AKT was assessed by Western blotting of (A) liver, (B) muscle, (C) adipose, and (D) heart tissue lysates from mice that were fasted overnight and then refed for 45 min. Additional western blots included in the quantification are shown in Fig. S2B. Young refers to 6‐month‐old males and females (10 males, 5 females), Middle refers to 24‐month‐old males and 22‐month‐old females (10 males, 5 females), and Old refers to 30‐month‐old males and 26‐month‐old females (8 males, 4 females). Quantification of AKT T308 and S473 are relative to AKT (**P < 0.01, *P < 0.05, #*P *< 0.09, two‐tailed *t*‐test, error bars indicate standard error).

We also observed significant differences in the phosphorylation of AKT S473 and T308 between males and females in muscle and adipose tissues, and we saw significant differences between aged males and females in liver and heart. In males, phosphorylation of the mTORC2 substrate AKT S473 after feeding was not altered with age in liver, adipose, or heart tissue, and showed a small decrease in Old male muscle (Fig. 4). However, in females AKT S473 was significantly increased in Old female muscle (Fig. 4B), and also increased in Old female heart (Fig. 4D).

AKT T308, the PDK1 site, decreased like AKT S473 in male muscle, but by a greater amount and in both Middle‐aged and Old mice. Surprisingly, while AKT S473 was increased in Old female muscle, AKT T308 was decreased by 50% (Fig. 4B). While mTORC2 signaling was not affected by aging in either male or female adipose tissue, we saw an age‐associated increase in AKT T308 phosphorylation in both Middle‐aged and Old male adipose Tissue, and an age‐associated decrease in female Old adipose tissue (Fig. 4C). Finally, AKT T308 was significantly increased in Old female heart (Fig. 4D).

### Sex specific differences in mTOR signaling in young mice

**Figure 5:**
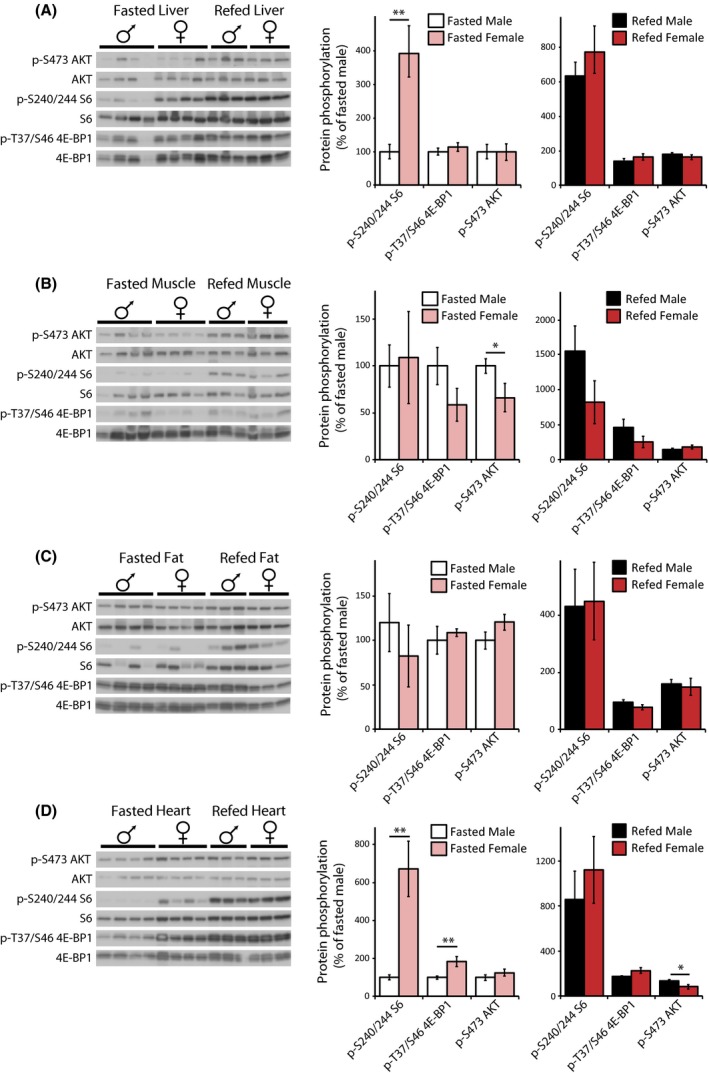
mTOR activity in male and female young C57BL/6J.Nia mice. (A–D) The phosphorylation of S6, 4E‐BP1, and AKT was assessed by Western blotting of (A) liver, (B) muscle, (C) adipose, and (D) heart tissue from mice that were either fasted overnight (Fasted) or fasted overnight and then refed for 45 min (Refed). Additional western blots included in the quantification are shown in Fig. S3. Quantification of S6 S240/S244 is relative to S6, 4E‐BP1 T37/S46 is relative to 4E‐BP1 and AKT S473 is relative to AKT (**P < 0.003, *P < 0.05, two‐tailed *t*‐test, error bars indicate standard error).

To determine how mTOR signaling might differ between sexes, we compared mTOR signaling in young (6 months old) male and female liver, muscle, adipose and heart tissue, examining both the fasted and refed state (Fig. 5). We observed a dramatic difference in S6 phosphorylation between the sexes in liver (Fig. 5A) and heart (Fig. 5D), with four‐fold higher S6 phosphorylation in fasted female liver than in male liver, and six‐fold higher S6 phosphorylation in fasted female heart than in male heart. No such difference was observed in muscle or adipose tissue, and S6 phosphorylation in the refed state was similar between males and females. Interestingly, consistent with generally higher mTORC1 signaling in fasted females, 4E‐BP1 phosphorylation was also elevated in fasted female hearts than in male hearts (Fig. 5D). Phosphorylation of AKT S473 was also slightly decreased in fasted female muscle and refed female hearts relative to male muscle or heart, respectively.

### Effect of aging on the transcriptome of aging C57BL/6J.Nia mice

Studies examining the aging transcriptome of rats and humans have reported a transcriptional downregulation of mTOR signaling during aging (Linford *et al*., 2007; Harries *et al*., 2012). Several studies, most notably the groundbreaking AGEMAP (Atlas of Gene Expression in Mouse Aging Project) study, have examined the effect of age on gene expression in C57BL/6J.Nia mice (Zahn *et al*., 2007) in tissues including liver, muscle, and heart. Notably, neither the AGEMAP study, nor a study published by Pearson *et al*. (2008) comparing the effect of dietary interventions on the aging C57BL/6J.Nia male transcriptome, identified the mTOR pathway as significantly affected (Zahn *et al*., 2007; Pearson *et al*., 2008).

Our knowledge of the mTOR signaling pathway has significantly increased since 2007, and we therefore decided to re‐analyze the AGEMAP and Pearson *et al*. gene expression data from liver, muscle, and heart tissue using GSEA (gene set enrichment analysis) (Mootha *et al*., 2003; Subramanian *et al*., 2005); unfortunately, adipose gene expression information was not available from the AGEMAP study, and an insufficient number of samples were available from the Pearson *et al*. study for GSEA. We utilized 25 gene sets for mTOR signaling from the Molecular Signature Database (Broad Institute). With a conservative FDR (false discovery rate) of 0.05, none of the gene sets examined were significantly enriched in the liver, muscle, or heart of either male or female mice with aging (Table S1, Supporting information). We did observe an enrichment of two gene sets in the Pearson *et al*. study, but only in the liver (Table S2).

### The trajectory of mTOR signaling with age may differ by strain and species

To determine whether the same changes in mTOR signaling occur in other mouse strains, we analyzed preserved female HET3 livers from a previous study (Lamming *et al*., 2013). The female HET3 mice were sacrificed after an overnight fast and 45 min of refeeding, similarly to the mice in Figs 3 and 4. While female C57BL/6J.Nia mice showed no change in the phosphorylation of S6 with age (Fig. 3A), we observed a 50% increase in S6 phosphorylation in the livers of Old female HET3 mice (Fig. S4A, Supporting information). Similarly, while Old female C57BL/6J.Nia mouse livers showed increased phosphorylation of AKT T308 (Fig. 4A), Old female HET3 livers had a trend toward decreased phosphorylation (Fig. S4A).

We proceeded to analyze livers from male DBA/2 mice and the livers, heart and muscle of male F344 Fisher Rats obtained from the NIA Aged Rodent Tissue Bank. These mice and rats were sacrificed in the morning following *ad libitum* feeding overnight. No differences in S6, 4E‐BP1, or AKT phosphorylation were observed in DBA/2 mice (Fig. S6A). In F344 rats, we observed no changes in mTORC1 signaling with age in liver (Fig. S4B), but observed significantly increased phosphorylation of S6 S240/S44 in 28‐month‐old rat muscle (Fig. S4C), and observed a trend toward decreased phosphorylation of 4E‐BP1 T37/S46 phosphorylation in 24‐ and 28‐month‐old adipose tissue (Fig. S4D). The phosphorylation of both AKT S473 and T308 increased with age in rat liver and adipose tissue (Fig. S4B,D), but in muscle we observed no change in AKT S473 and an age‐associated decrease in AKT T308 phosphorylation.

## Discussion

Recently, numerous studies have reported altered mTOR signaling with age, with the majority suggesting that mTORC1 signaling increases with age in a variety of tissues and cell types (Table 1). However, many of these studies utilized relatively young mice of a single sex, and analyzed only a single readout of mTORC1 signaling in a single tissue. Here, we have examined the phosphorylation of S6K1 T389, S6 S240/S244, and 4E‐BP1 T37/S46, as well as AKT T308 and S473 in four different tissues, utilizing mice that are roughly comparable to 25‐ to 30‐year‐old, 56‐ to 69‐year‐old, and 80‐year‐old humans (Flurkey *et al*., 2007). Further, we utilized well‐studied C57BL/6J.Nia mice, the lifespan of which can be extended by rapamycin (Fok *et al*., 2014), and in which rapamycin can rejuvenate both the aging heart and HSCs (Chen *et al*., 2009; Dai *et al*., 2014).

**Figure 6:**
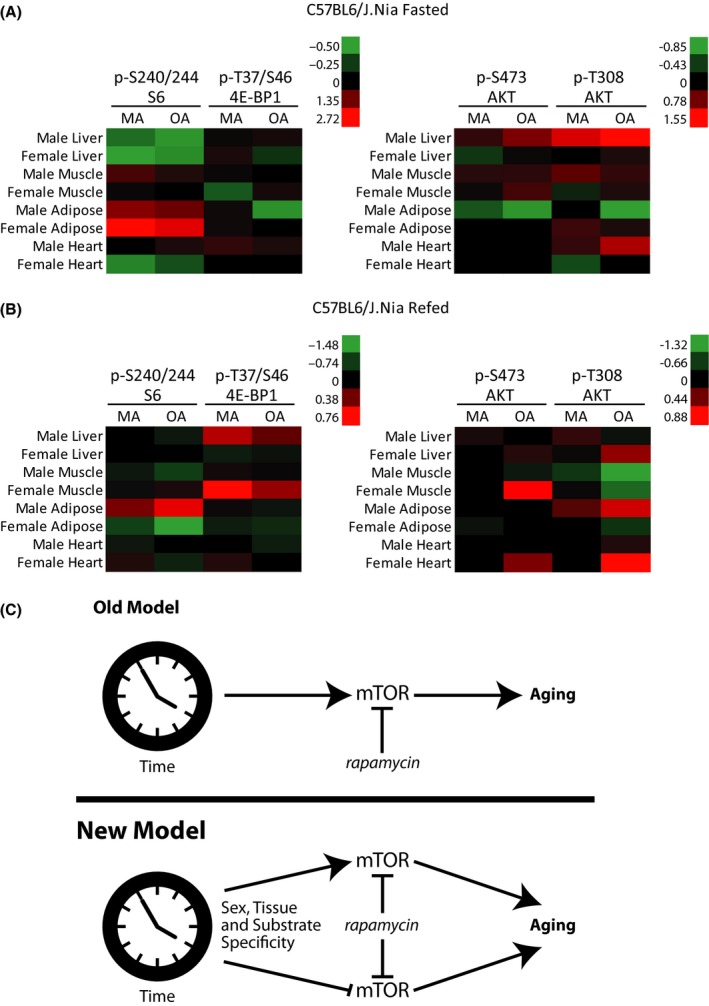
A heat map summarizing the average fold‐change (log_2_) in the phosphorylation of specific residues in Middle‐aged (MA) and Old (OA) C57BL/6J.Nia mice vs. Young (6 months old) control mice. A color key is provided to the right of each heat map, numbers indicate the log2 fold‐change relative to the Young (6 months old) mice. (C) One conceptual model (‘Old Model’) is that increasing chronological age induces a pathological increase in mTOR activity, which in turn promotes the pathophysiology of aging. In this model, rapamycin might act to promote lifespan by blocking this age‐associated pathological increase in mTOR signaling. Our findings support a different model (‘New Model’), in which the effect of age on mTOR activity varies by substrate, tissue, and sex, and in which aging does not lead to a generalized increase in mTOR signaling. In this model, rapamycin acts to decrease mTOR signaling generally.

When presented using heat maps (Fig. 6A,B), it is immediately apparent that the trajectory of mTORC1 signaling with age is highly dependent upon the tissue involved and that the phosphorylation status of S6 is much more variable than the phosphorylation of 4E‐BP1 (Fig. 6A). We also observed sex‐based differences in the trajectory of mTOR signaling with age, which may help to explain the sexually dimorphic impact of rapamycin on lifespan (Miller *et al*., 2014). In the fasted state, sex‐based differences in S6 phosphorylation in the heart are observed (Fig. 6A), while in the refed state (Fig. 6B), sex‐based differences in both adipose S6 phosphorylation and muscle 4E‐BP1 phosphorylation are apparent.

In addition to sex‐based differences in the trajectory of mTOR signaling with age, we observed significantly higher mTORC1 signaling in young fasted female liver and heart than in males (Fig. 5A,D). While the basis for this difference is unknown, higher mTORC1 signaling in female C57BL/6J.Nia mice correlates with both their slightly shorter lifespan relative to males, and to the greater increase in lifespan female mice show following treatment with rapamycin (Turturro *et al*., 1999; Fok *et al*., 2014). The differences observed here may also contribute to the sexually dimorphic impact of *S6K1* deletion on lifespan (Selman *et al*., 2009).

In the refed state, where we were able to consistently detect both phosphorylated S6K1 T389 and phosphorylated S6 S240/244 in most tissues, we observed a significant divergence between these two readouts of mTORC1 activity in muscle (Fig. 3B). It is most likely that this is due to different kinetics in the phosphorylation of these two sites, as S6K1 responds much more quickly to changes in mTORC1 activity (Rosner & Hengstschlager, 2010). Other possibilities include alterations in the levels of protein phosphatases such as protein phosphatase 2A or other post‐translational factors that might impact S6K1 activity in aged mice or differentially impact S6K1 or S6 phosphorylation. Understanding how the activity of protein phosphatases may change with age is an important unanswered question.

Our results do not correlate perfectly with previous studies on the changes of mTOR signaling with age (Table 1). However, the tightness of our age groups, our separate analysis of the sexes, the uniform genetic background of our mice, and the relatively large number of mice analyzed provide us with significant confidence in our findings. Our GSEA analysis of the transcriptome of aging C57BL/6J.Nia mice corroborates our assessment of only relatively small changes in mTOR signaling with age in liver, muscle, and heart. Differences in genetic background or diet may contribute to variability between studies, and indeed, we do not know if all the mouse strains used in Table 1 respond similarly to dietary rapamycin. We were also unable to examine how the effect of dietary rapamycin on mTOR signaling might change with age due to insufficient supply of aged mice. However, we note that our study represents the first extensive analysis of age‐associated changes in mTOR signaling in a mouse strain (C57BL/6J.Nia) that is demonstrated to live longer following rapamycin treatment (Fok *et al*., 2014). Future longitudinal studies using advanced techniques (e.g., *in vivo* imaging) to track how mTOR signaling changes with age and rapamycin in individual mice that are permitted to live a natural lifespan may shed light on how mTOR signaling at the individual level correlates with lifespan.

In our initial conceptual model, we theorized that mTOR signaling might naturally increase with time, driving the pathology of aging (Fig. 6C, Old Model). Our results demonstrate that this is an overly simple model, and that the effect of time on mTOR signaling actually varies by sex, tissue and substrate (Fig. 6C, New Model). Rapamycin treatment will inhibit mTOR in all tissues, regardless of the increase or decrease in the tissue of mTOR signaling with age, promoting longevity (Fig. 6C, New Model). It is important to note that even the lower level of mTOR signaling found in tissues in which mTOR decreases with age could still ‘be inappropriately high for an aging organism’ (Leontieva *et al*., 2014), and contribute to the pathophysiology of aging. The mechanistic basis for the varying impact of age and sex on mTOR signaling in different tissues is an important unanswered question that remains to be addressed.

We conclude that aging does not result in a generalized increase in mTOR signaling in C57BL/6J.Nia mice, a strain in which dietary rapamycin significantly extends lifespan and rejuvenates the aging heart (Dai *et al*., 2014; Fok *et al*., 2014). We do observe age‐associated increases in mTOR signaling in specific tissues of C57BL/6J.Nia mice, and the beneficial effects of rapamycin on longevity in this strain may be due in part to the blunting of age‐associated increases in mTOR signaling in specific tissues such as adipose and HSCs (Chen *et al*., 2009). It remains to be determined whether mouse strains with greater age‐associated increases in mTORC1 signaling respond even more favorably to rapamycin. Finally, we conclude that the trajectory of mTOR signaling with age varies not only by tissue, but also by sex, the substrate considered, and may also vary between mouse strains and between different species.

## Experimental procedures

### Materials

Antibodies to phospho‐Akt S473 (4060), phospho‐Akt T308 (9275), Akt (4691), phospho‐p70 S6 kinase (9234), p70 S6 kinase (2708), phospho‐S6 ribosomal protein (2215), S6 ribosomal protein (2217), p‐4EBP1 T37/46 (2855), and total 4EBP1 (9452) were from Cell Signaling Technology. Protease and phosphatase inhibitor cocktail tablets were from Fisher. Other chemicals were purchased from Sigma unless noted. Glucose measurements were performed using a Bayer Contour blood glucose meter and test strips. Mouse Insulin ELISA kits were purchased from Crystal Chem. 2.0‐mL Tough Tubes with Caps (13119‐500) and 1.4‐mM ceramic beads (13113‐325) were purchased from Mo‐Bio Laboratories, Carlsbad, CA.

### Immunoblotting

Cells and tissue samples were lysed in cold RIPA buffer supplemented with phosphatase inhibitor and protease inhibitor cocktail tablets. Tissues were lysed in RIPA buffer as previously described (Lamming *et al*., 2012) using a FastPrep 24 (M.P. Biomedicals) with bead‐beating tubes and ceramic beads (Mo‐Bio Laboratories), and then centrifuged for 10 min at 15 000 rpm. Protein concentration was determined by Bradford (Pierce Biotechnology). 20 μg protein was separated by SDS–PAGE (sodium dodecyl sulfate–polyacrylamide gel electrophoresis) on 8%, 10%, or 16% resolving gels (Life Technologies/ThermoFisher) and transferred to PVDF membrane. Depending upon the strength of the antibody and the signal, which varied by tissue, antibody, and feeding state, the membranes were either stripped and reprobed for total antibody, or duplicate gels were run and separate blots were probed. Duplicate gels were always run for phosphorylated S6 and total S6, and phosphorylated 4E‐BP1 and total 4E‐BP1. In contrast, phosphorylated Akt S473 and Akt T308 were always run on duplicate gels, and Akt T308 was usually stripped and reprobed for total AKT. Phosphorylated S6K1 was usually stripped and reprobed for total S6K1.

### Quantification of immunoblots

Imaging was performed using a GE ImageQuant LAS 4000 imaging station, and images were captured as 16‐bit TIFs. Quantification was performed by densitometry using NIH ImageJ software, and samples in which no total protein was detected were excluded from the analysis of both the phosphorylated and total protein. Data from each immunoblot were normalized by dividing the value of each lane by the average of the control values (6‐month‐old mice) for the same immunoblot.

### Gene set enrichment analysis

We used the Java desktop application of GSEA (www.broadinstitute.org/gsea/index.jsp) for gene set enrichment analysis. Input files were prepared as described by the GSEA user guide. The mTOR pathway gene sets we used were obtained from the Molecular Signatures Database (MSigDB) and are listed in Table S3. The GSEA application was executed using default parameters as recommended by (Subramanian *et al*., 2005).

### Animals and treatments

Animal studies were approved by the Institutional Animal Care and Use Committee of the University of Wisconsin‐Madison and the William S. Middleton Memorial Veterans Hospital, Madison WI. C57BL/6J.Nia mice maintained with *ad libitum* access to NIH 31 diet with the ages and sexes specified were obtained from the NIA Aged Rodent Colony and then housed locally for 2–4 weeks with *ad libitum* access to LabDiet 5001 diet prior to euthanasia. Mice were fasted overnight and sacrificed approximately 16 h later, or refed at that time for 45 min and then sacrificed. Mice sacrificed in the fasted state: 6‐month‐old males and females (10 males, 5 females), 24‐month‐old males and 22‐month‐old females (10 males, 5 females), 30‐month‐old males and 26‐month‐old females (8 males, 4 females). An equal number of mice were sacrificed after refeeding for 45 min. Several additional mice in the Middle‐age and Old‐age group were sacrificed and all tissues excluded due to obvious metastatic cancer. Tissue obtained from the NIA Aged Rodent Tissue Bank was harvested from mice in the morning following *ad libitum* overnight feeding; all tissues obtained and analyzed were tumor free.

## Funding

The Lamming lab is supported by a K99/R00 Pathway to Independence Award to DWL from the National Institute of Health/NIA (AG041765), as well as startup funds from the UW‐Madison School of Medicine and Public Health and the UW‐Madison Department of Medicine. KAC was supported in part by a Research Supplement to Promote Diversity in Health‐Related Research (AG041765‐04S1). IO is supported by the University of Wisconsin Carbone Cancer Center Support Grant P30 CA014520 and the Clinical and Translational Science Award (CTSA) program, through the NIH National Center for Advancing Translational Sciences (NCATS), grant UL1TR000427. This work was supported using facilities and resources from the William S. Middleton Memorial Veterans Hospital. This work does not represent the views of the Department of Veterans Affairs or the United States Government.

## Conflict of interest

None declared.

## Supporting information


**Fig. S1** Additional westerns used for the quantification of phosphorylated proteins graphs in Figs 1 and 2.

**Fig. S2** Additional westerns used for the quantification of phosphorylated proteins graphs in Figs 3 and 4.

**Fig. S3** Additional westerns used for the quantification of phosphorylated proteins graphs in Fig. 5.

**Fig. S4** mTOR signaling in female HET3 mice and F344 male rats. (A–D) Western blots for mTOR pathway substrates and readouts in (A) the livers of genetically heterogeneous HET3 female mice at 8 and 23 months of age fasted overnight and then refed for 45 min; and (B) liver, (C) muscle, and (D) adipose tissue of male F344 rats at 4, 24, and 28 months of age obtained from the NIA Aged Rodent Tissue Bank. Additional westerns included in the quantification are shown in Fig. S5. Quantification of each phosphorylated substrate is relative to their respective total protein (**P < 0.01, *P < 0.05, #*P *< 0.09, two‐tailed *t*‐test, error bars indicate standard error).

**Fig. S5** Additional westerns used for the quantification of phosphorylated proteins graphs in Fig. S4.

**Fig. S6** mTOR signaling in different mouse strains and in rats. (A) mTOR signaling in the livers of 4‐month old (young) 21‐month old (old) DBA/2 mice obtained from the NIA Aged Rodent Tissue Bank. (B) A heat map summarizing the average fold‐change (log_2_) in the phosphorylation of specific residues in Middle‐aged (MA) and Old (OA) mice or rats vs. Young (6‐month old) control mice or rats. A color key is provided to the right of each heat map.

**Table S1** GSEA of AGEMAP data (Zahn *et al*., 2007) from liver, muscle, and heart using 23 mTOR‐related gene sets. The false discovery rate is shown for each gene set.

**Table S2** GSEA of Pearson *et al*. (2008) data from liver, muscle, and heart using 23 mTOR‐related gene sets. The FDR (false discovery rate) is shown for each gene set; gene sets with an FDR* *< 0.05 are highlighted in yellow.

**Table S3** List of gene sets used for GSEA analysis.
