# Supplementary figures and images for: Sex‐ and tissue‐specific changes in mTOR signaling with age in C57BL/6J mice
Source: Aging Cell. 2015 Nov 24;15(1):155–66. doi: 10.1111/acel.12425 (PMC4717274; doi:10.1111/acel.12425)

Figure S1

A)

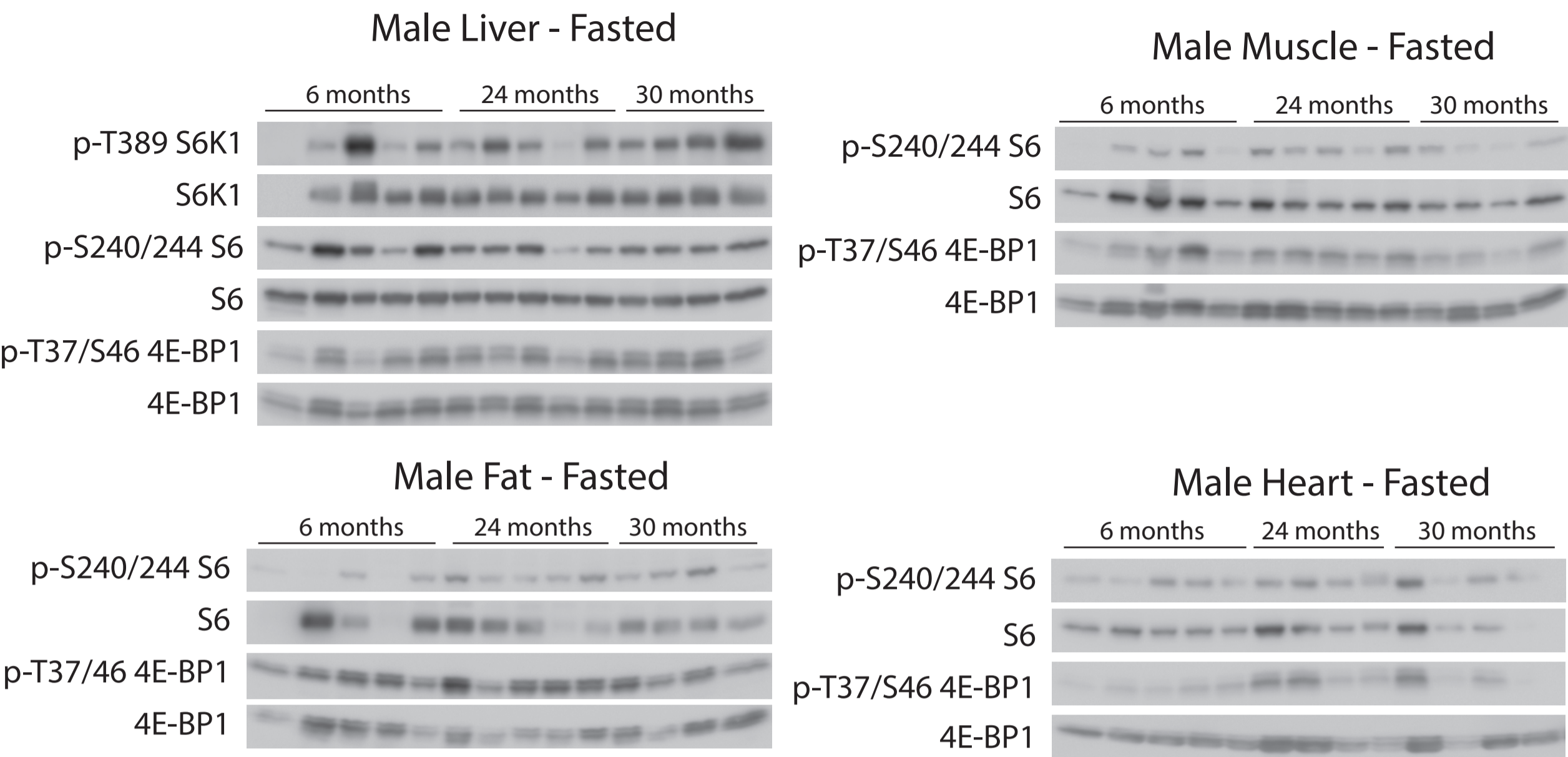

B)

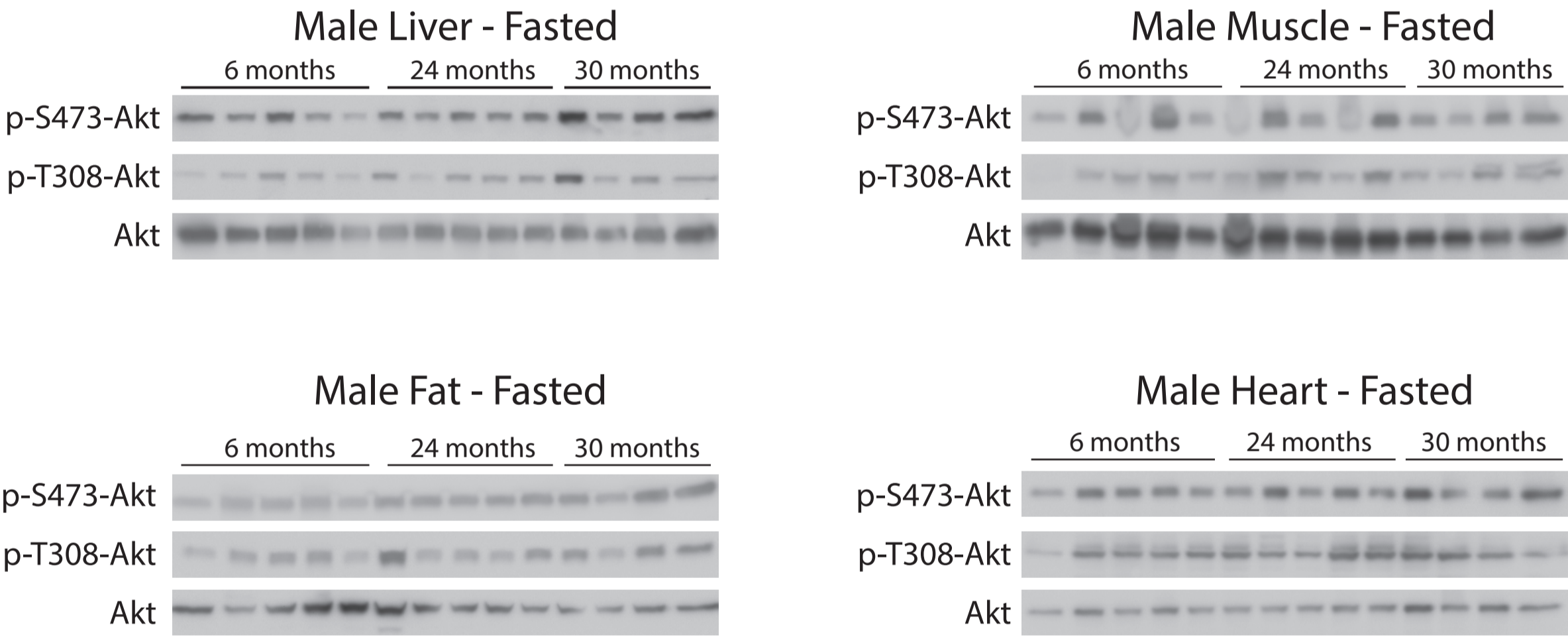

Supplement: Supplementary file 1 — Fig. S1 Additional westerns used for the quantification of phosphorylated proteins graphs in Figs 1 and 2. [file ACEL-15-155-s001.pdf]

Figure S2

A)

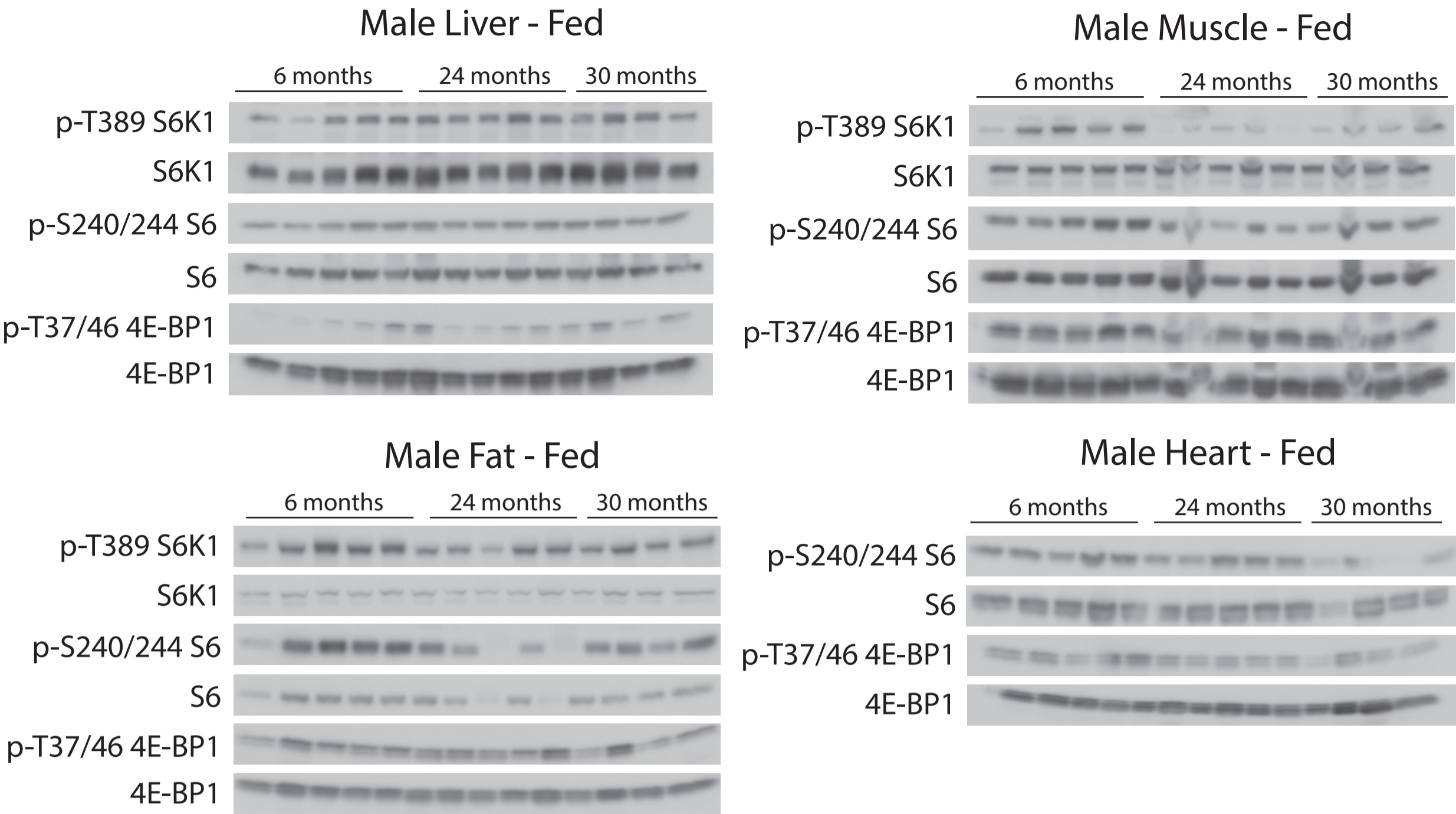

B)

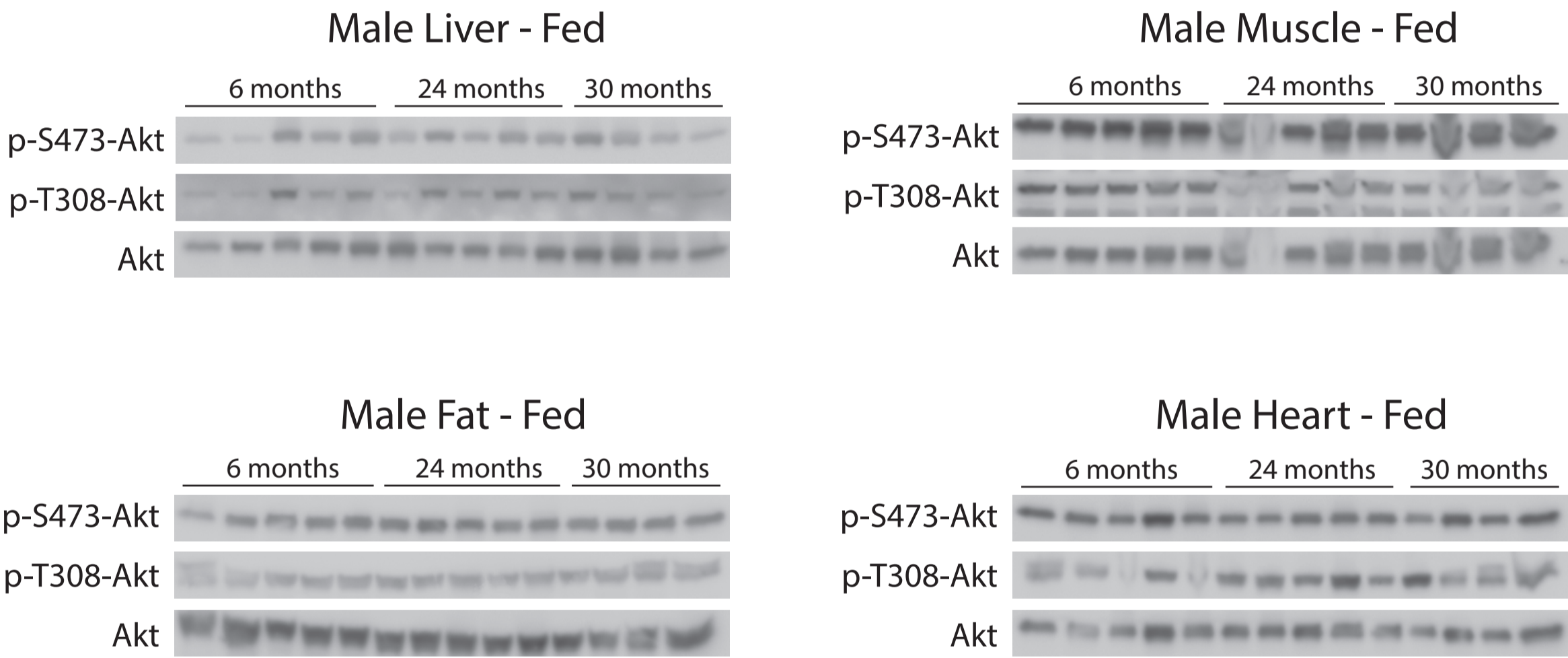

Supplement: Supplementary file 2 — Fig. S2 Additional westerns used for the quantification of phosphorylated proteins graphs in Figs 3 and 4. [file ACEL-15-155-s002.pdf]

Figure S3

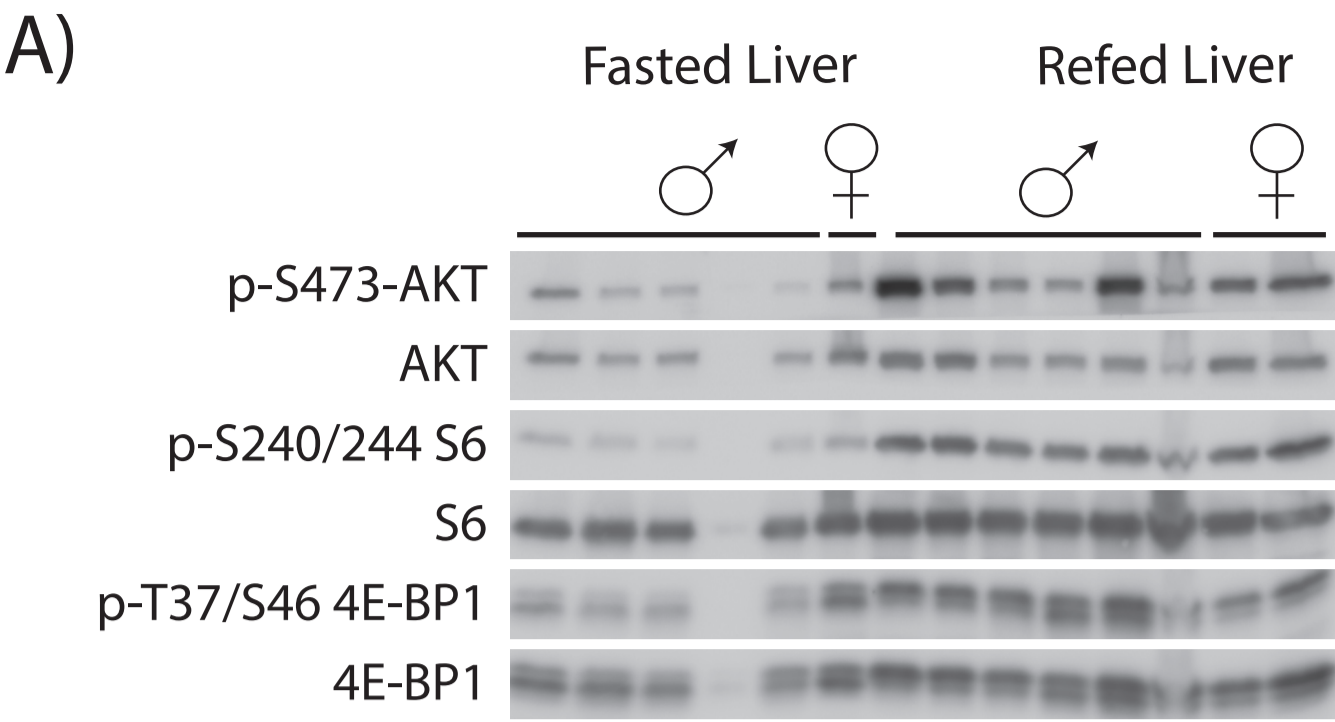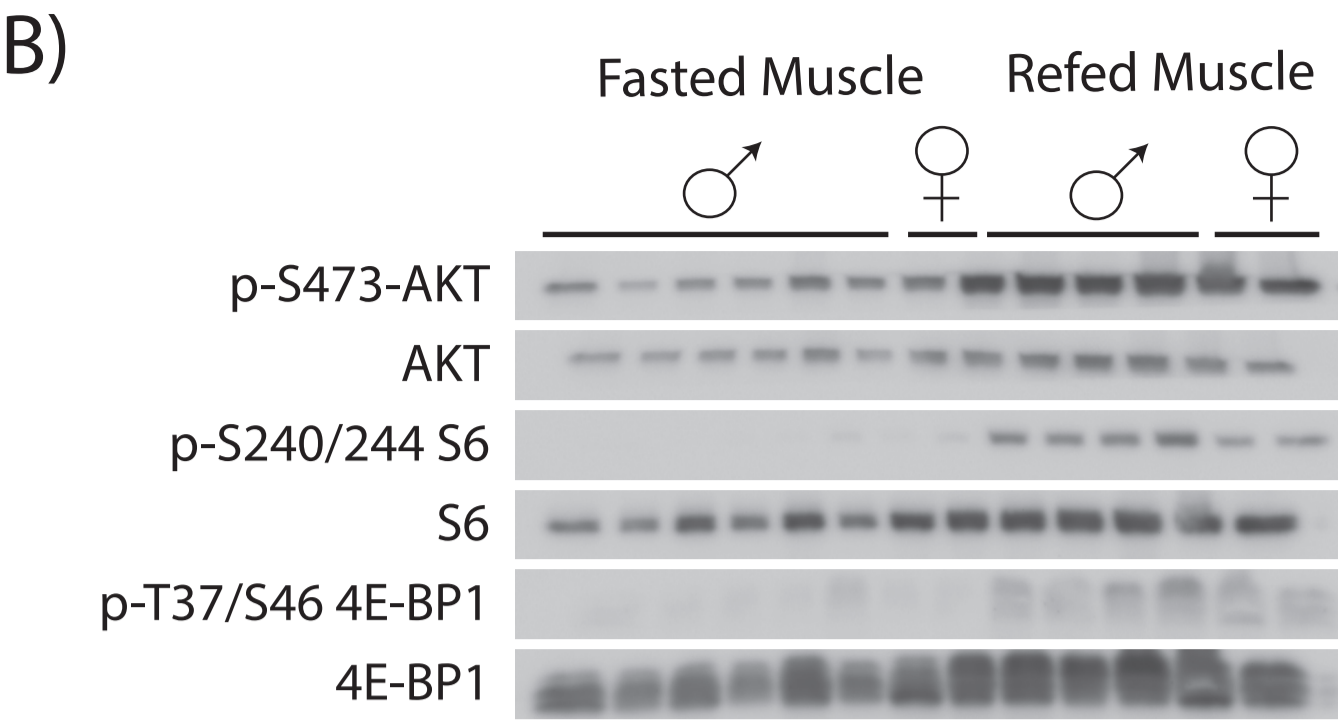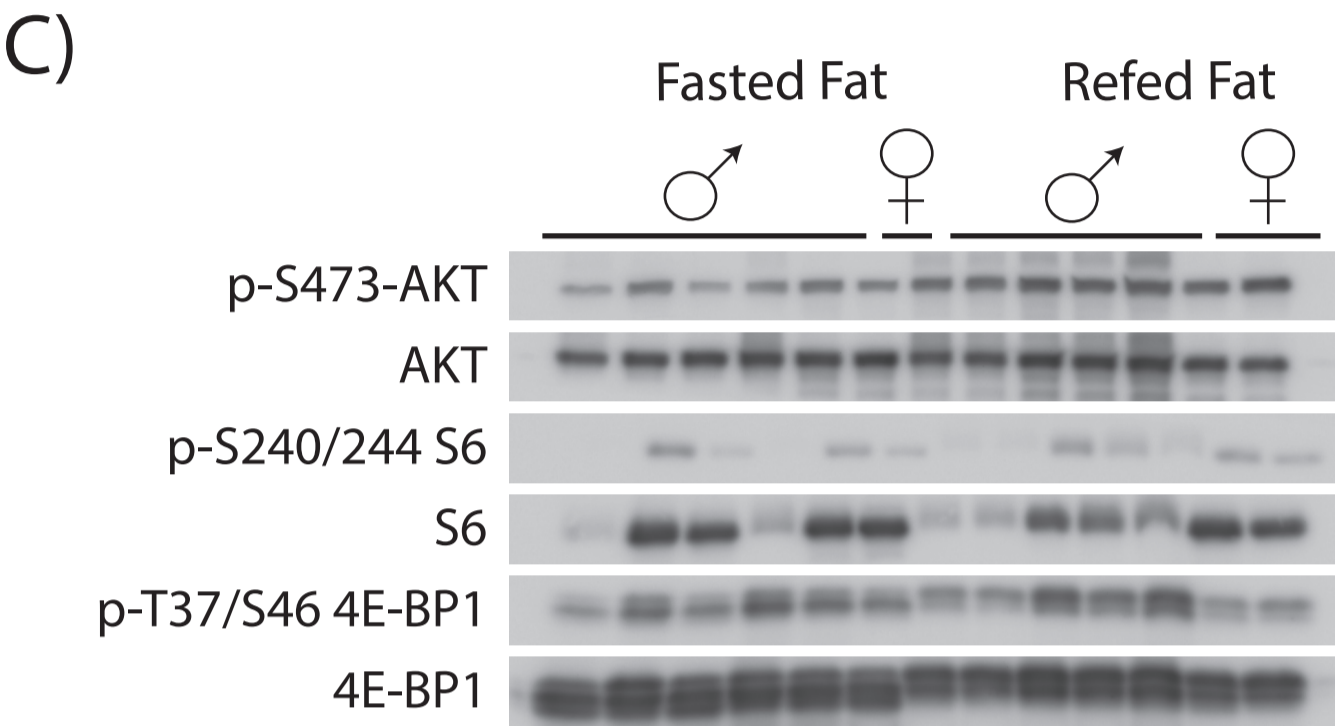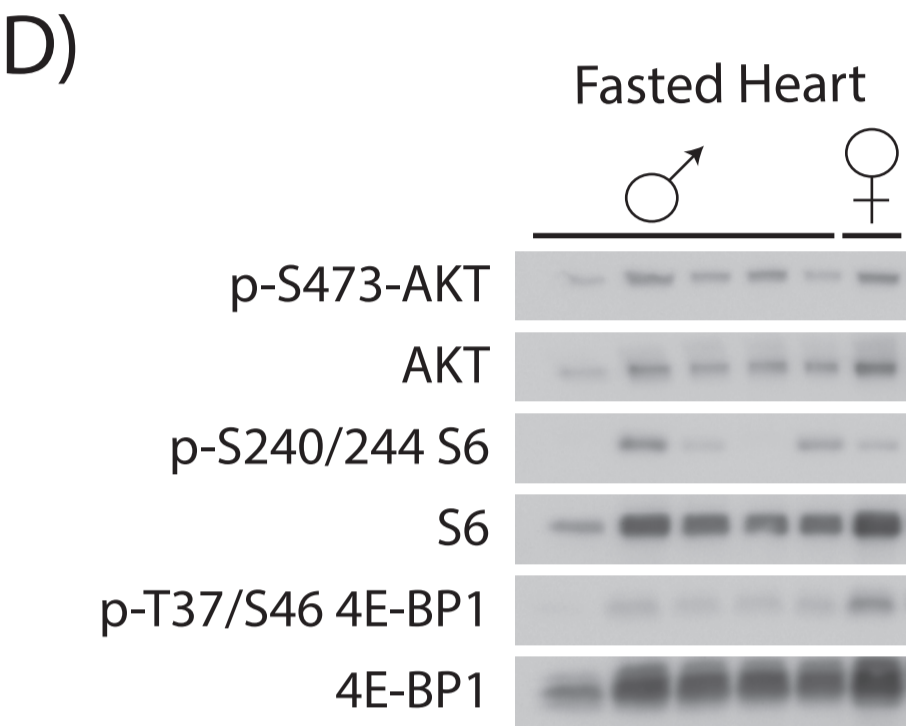

Supplement: Supplementary file 3 — Fig. S3 Additional westerns used for the quantification of phosphorylated proteins graphs in Fig. 5. [file ACEL-15-155-s003.pdf]

Figure S4

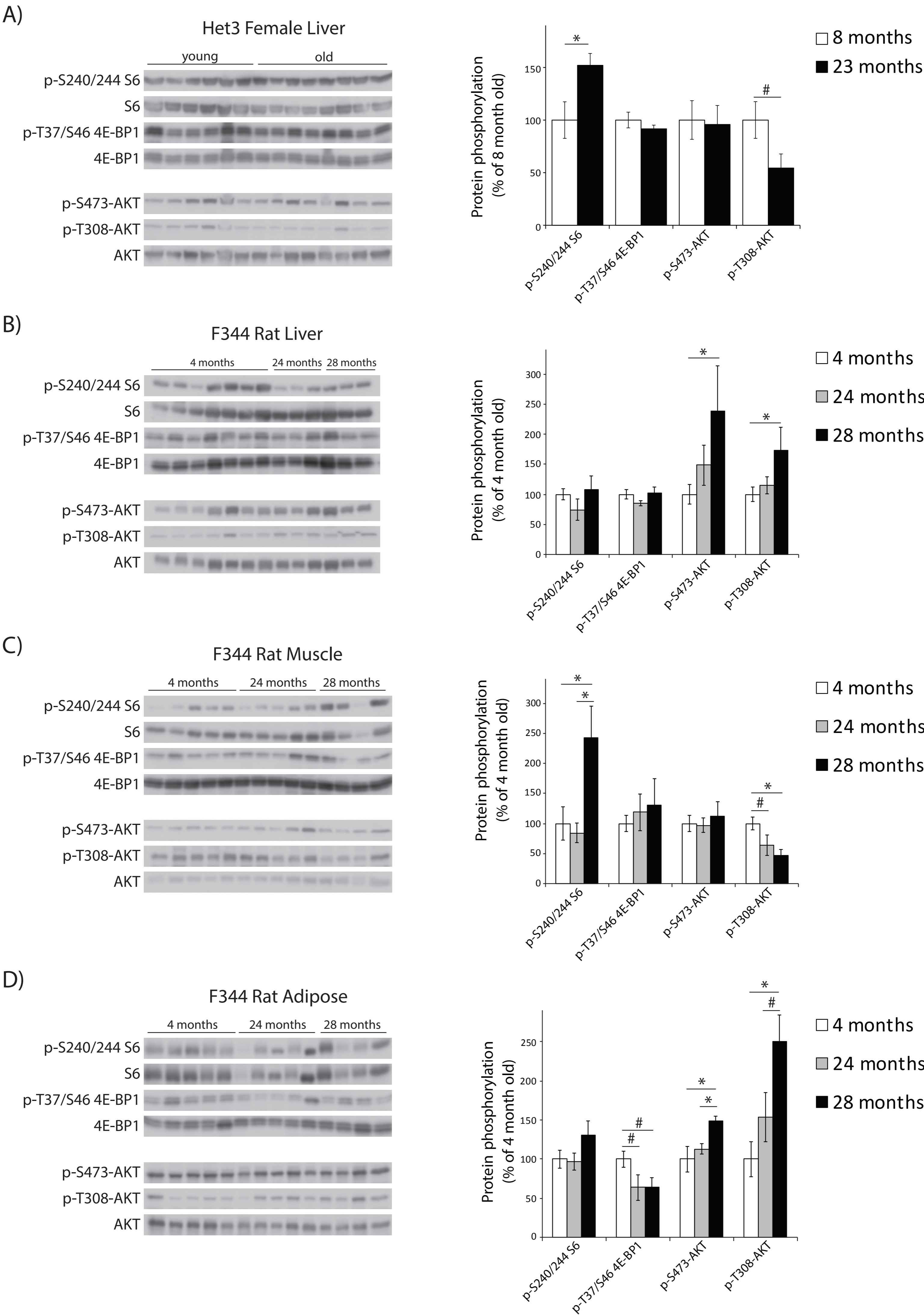

Supplement: Supplementary file 4 — Fig. S4 mTOR signaling in female HET3 mice and F344 male rats. (A–D) Western blots for mTOR pathway substrates and readouts in (A) the livers of genetically heterogeneous HET3 female mice at 8 and 23 months of age fasted overnight and then refed for 45 min; and (B) liver, (C) muscle, and (D) adipose tissue of male F344 rats at 4, 24, and 28 months of age obtained from the NIA Aged Rodent Tissue Bank. Additional westerns included in the quantification are shown in Fig. S5. Quantification of each phosphorylated substrate is relative to their respective total protein (**P < 0.01, *P < 0.05, #P < 0.09, two‐tailed t‐test, error bars indicate standard error). [file ACEL-15-155-s004.pdf]

Figure S5

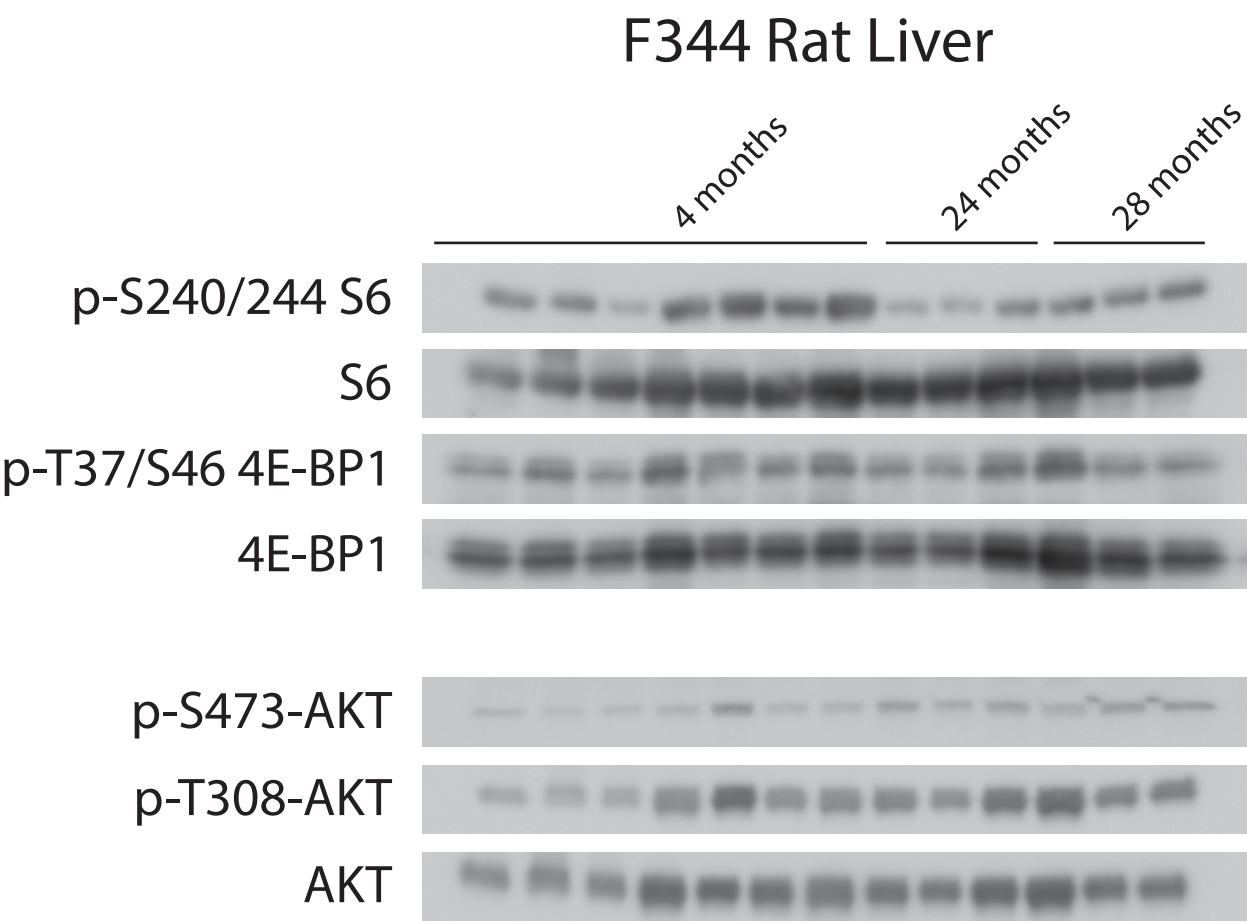

Supplement: Supplementary file 5 — Fig. S5 Additional westerns used for the quantification of phosphorylated proteins graphs in Fig. S4. [file ACEL-15-155-s005.pdf]

Figure S6

A)

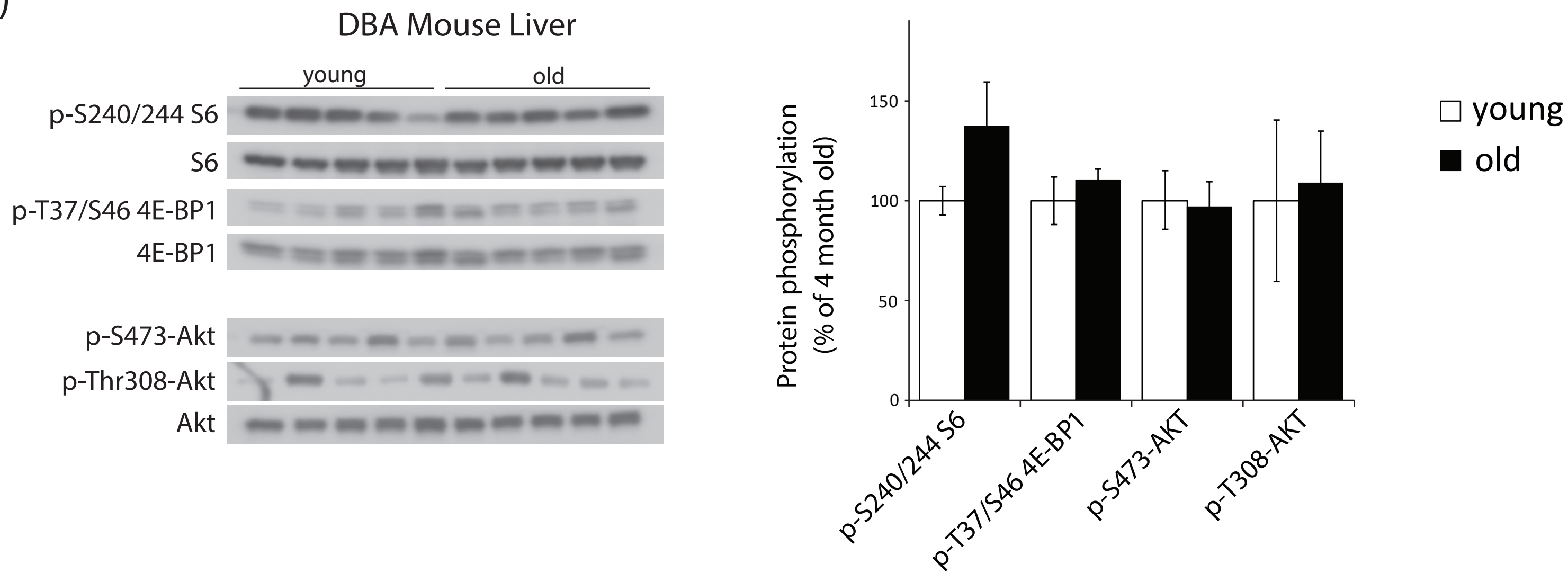

B)

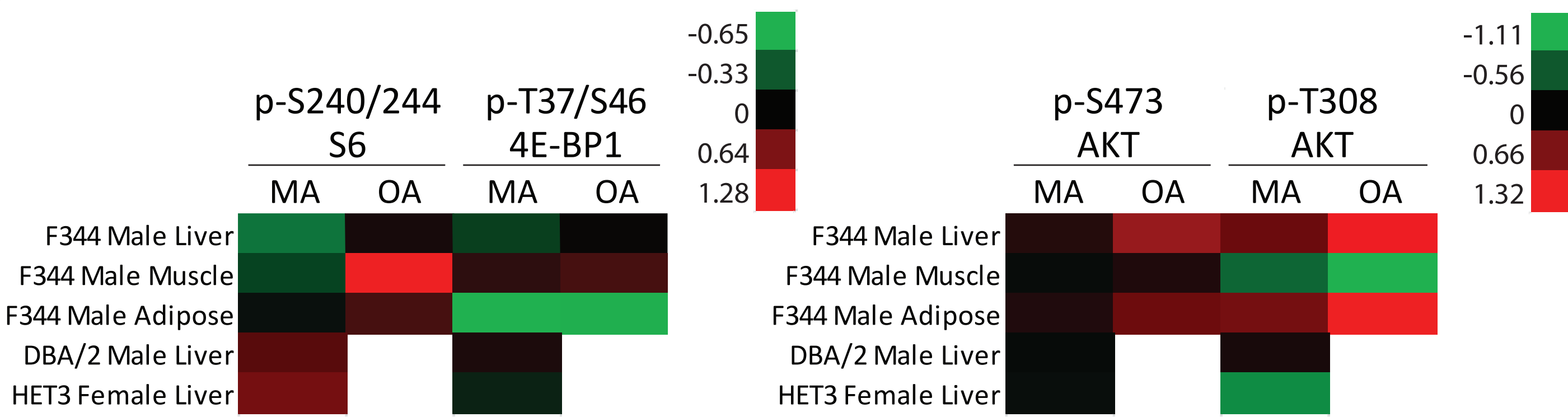

Supplement: Supplementary file 6 — Fig. S6 mTOR signaling in different mouse strains and in rats. (A) mTOR signaling in the livers of 4‐month old (young) 21‐month old (old) DBA/2 mice obtained from the NIA Aged Rodent Tissue Bank. (B) A heat map summarizing the average fold‐change (log2) in the phosphorylation of specific residues in Middle‐aged (MA) and Old (OA) mice or rats vs. Young (6‐month old) control mice or rats. A color key is provided to the right of each heat map. [file ACEL-15-155-s006.pdf]
