# Supplementary material for: Sex‐ and tissue‐specific changes in mTOR signaling with age in C57BL/6J mice
Source: Aging Cell. 2015 Nov 24;15(1):155–66. doi: 10.1111/acel.12425 (PMC4717274; doi:10.1111/acel.12425)
Supplement: Supplementary file 7 — Table S1 GSEA of AGEMAP data (Zahn et al., 2007) from liver, muscle, and heart using 23 mTOR‐related gene sets. The false discovery rate is shown for each gene set. [file ACEL-15-155-s007.pdf]

Table S1

	Liver F	Liver F	Liver M	Liver M	Muscle F	Muscle F	Muscle M	Muscle M	Heart F	Heart F	Heart M	Heart M
**Genesets**	young	aged	young	aged	young	aged	young	aged	young	aged	young	aged
BIOCARTA_IGF1MTOR_PATHWAY		1.000		0.622		0.808		0.349	0.893			1.000
BIOCARTA_MTOR_PATHWAY		0.826		0.458		0.605		0.859	0.841		0.937	
BOYLAN_MULTIPLE_MYELOMA_C_CLUSTER_UP		0.972		0.796	1.000		1.000			1.000	0.250	
BOYLAN_MULTIPLE_MYELOMA_C_UP			1.000		1.000		0.930		0.928		0.366	
BYSTRYKH_HEMATOPOIESIS_STEM_CELL_QTL_TRANS	1.000		1.000		1.000		1.000		0.967		0.335	
BRUINS_UVC_RESPONSE_VIA_TP53_GROUP_C	1.000		1.000			1.000		0.916	0.972		0.259	
CREIGHTON_AKT1_SIGNALING_VIA_MTOR_DN	1.000			0.996		0.985	0.077			0.883		1.000
CREIGHTON_AKT1_SIGNALING_VIA_MTOR_UP		0.953		0.579		0.981		0.190	1.000			0.962
GSE17721_LPS_VS_GARDIQUIMOD_6H_BMDM_UP	1.000		1.000		1.000		0.684		1.000			1.000
GSE17721_LPS_VS_POLYIC_6H_BMDM_DN		0.857		0.647		0.572		1.000		0.883	1.000	
GSE17721_PAM3CSK4_VS_GADIQUIMOD_6H_BMDM_UP	1.000		1.000		1.000		0.863		0.978		0.806	
GSE19825_NAIVE_VS_IL2RAHIGH_DAY3_EFF_CD8_TCELL_DN		0.915	1.000		1.000			0.859	1.000			1.000
GSE19825_NAIVE_VS_IL2RALOW_DAY3_EFF_CD8_TCELL_DN		0.637		0.994		1.000	0.930			0.982		1.000
IVANOVA_HEMATOPOIESIS_STEM_CELL_AND_PROGENITOR		1.000		0.574		0.634		0.961		1.000	0.953	
IWANAGA_CARCINOGENESIS_BY_KRAS_PTEN_DN	1.000		1.000			1.000	0.997		0.874		0.778	
LEE_AGING_CEREBELLUM_DN	1.000		1.000		1.000		1.000		1.000			1.000
LEE_AGING_NEOCORTEX_DN	1.000			0.650		0.646		0.982		0.960	1.000	
MTOR-NIHMS-INCHIANTI		0.675		0.617		0.604		1.000	1.000		0.674	
MTOR-NIHMS-SAFHS		0.982		0.332		0.700		1.000	0.844		0.731	
REACTOME_CD28_DEPENDENT_PI3K_AKT_SIGNALING				0.329		0.743		0.056		1.000		1.000
WAKABAYASHI_ADIPOGENESIS_PPARG_BOUND_36HR			1.000		0.084		0.961		1.000			1.000
WAKABAYASHI_ADIPOGENESIS_PPARG_BOUND_8D		0.954		0.843		1.000		1.000	1.000		0.861	
