# Supplementary material for: Sex‐ and tissue‐specific changes in mTOR signaling with age in C57BL/6J mice
Source: Aging Cell. 2015 Nov 24;15(1):155–66. doi: 10.1111/acel.12425 (PMC4717274; doi:10.1111/acel.12425)
Supplement: Supplementary file 8 — Table S2 GSEA of Pearson et al. (2008) data from liver, muscle, and heart using 23 mTOR‐related gene sets. The FDR (false discovery rate) is shown for each gene set; gene sets with an FDR < 0.05 are highlighted in yellow. [file ACEL-15-155-s008.pdf]

Table S2

	Liver	Liver	Muscle	Muscle	Heart	Heart
**Genesets**	in SD_18	in SD_27	in SD_18	in SD_27	in SD_18	in SD_27
BIOCARTA_MTOR_PATHWAY	0.633		1.000		0.455	
BOYLAN_MULTIPLE_MYELOMA_C_CLUSTER_UP		0.324		0.362	0.830	
BOYLAN_MULTIPLE_MYELOMA_C_UP	0.873			0.883		0.877
BRUINS_UVC_RESPONSE_VIA_TP53_GROUP_C	0.791		0.937			0.689
CREIGHTON_AKT1_SIGNALING_VIA_MTOR_DN		0.030		0.726	0.179	
CREIGHTON_AKT1_SIGNALING_VIA_MTOR_UP		0.329	0.435			0.217
GSE17721_LPS_VS_GARDIQUIMOD_6H_BMDM_UP		0.292		0.353		0.417
GSE17721_LPS_VS_POLYIC_6H_BMDM_DN		0.046		0.504		0.254
GSE17721_PAM3CSK4_VS_GADIQUIMOD_6H_BMDM_UP	0.917			0.489		0.108
GSE19825_NAIVE_VS_IL2RAHIGH_DAY3_EFF_CD8_TCELL_DN		0.339	1.000			0.771
GSE19825_NAIVE_VS_IL2RALOW_DAY3_EFF_CD8_TCELL_DN		0.447	1.000			0.824
IVANOVA_HEMATOPOIESIS_STEM_CELL_AND_PROGENITOR	1.000		1.000		0.462	
IWANAGA_CARCINOGENESIS_BY_KRAS_PTEN_DN		0.337	1.000		0.581	
LEE_AGING_CEREBELLUM_DN		0.487		0.445	0.593	
LEE_AGING_NEOCORTEX_DN	1.000			0.947		0.777
MTOR-NIHMS-INCHIANTI		0.716		0.765		0.478
MTOR-NIHMS-SAFHS	0.864		1.000			0.453
REACTOME_CD28_DEPENDENT_PI3K_AKT_SIGNALING		0.310		0.901	0.662	
WAKABAYASHI_ADIPOGENESIS_PPARG_BOUND_36HR		0.674		0.550		0.424
WAKABAYASHI_ADIPOGENESIS_PPARG_BOUND_8D	1.000			0.315		0.284
