# Supplementary material for: Sex‐ and tissue‐specific changes in mTOR signaling with age in C57BL/6J mice
Source: Aging Cell. 2015 Nov 24;15(1):155–66. doi: 10.1111/acel.12425 (PMC4717274; doi:10.1111/acel.12425)
Supplement: Supplementary file 9 — Table S3 List of gene sets used for GSEA analysis. [file ACEL-15-155-s009.pdf]

Table S3

BIOCARTA_IGF1MTOR_PATHWAY
BIOCARTA_MTOR_PATHWAY
BOYLAN_MULTIPLE_MYELOMA_C_CLUSTER_UP
BOYLAN_MULTIPLE_MYELOMA_C_UP
BRUINS_UVC_RESPONSE_VIA_TP53_GROUP_C
BYSTRYKH_HEMATOPOIESIS_STEM_CELL_QTL_TRANS
CORRADETTI_MTOR_PATHWAY_REGULATORS_DN
CREIGHTON_AKT1_SIGNALING_VIA_MTOR_DN
CREIGHTON_AKT1_SIGNALING_VIA_MTOR_UP
GSE17721_LPS_VS_GARDIQUIMOD_6H_BMDM_UP
GSE17721_LPS_VS_POLYIC_6H_BMDM_DN
GSE17721_PAM3CSK4_VS_GADIQUIMOD_6H_BMDM_UP
GSE19825_NAIVE_VS_IL2RAHIGH_DAY3_EFF_CD8_TCELL_DN
GSE19825_NAIVE_VS_IL2RALOW_DAY3_EFF_CD8_TCELL_DN
IVANOVA_HEMATOPOIESIS_STEM_CELL_AND_PROGENITOR
IWANAGA_CARCINOGENESIS_BY_KRAS_PTEN_DN
LEE_AGING_CEREBELLUM_DN
LEE_AGING_NEOCORTEX_DN
mTORC2-liver
mTOR-NIHMS-InCHIANTI
mTOR-NIHMS-SAFHS
PILON_KLF1_TARGETS_DN
REACTOME_CD28_DEPENDENT_PI3K_AKT_SIGNALING
WAKABAYASHI_ADIPOGENESIS_PPARG_BOUND_36HR
WAKABAYASHI_ADIPOGENESIS_PPARG_BOUND_8D
